# Pyridin-4-ylmethanaminium perchlorate mono­hydrate

**DOI:** 10.1107/S2414314623004595

**Published:** 2023-05-26

**Authors:** Rüdiger W. Seidel, Tsonko M. Kolev

**Affiliations:** a Institut für Pharmazie, Martin-Luther-Universität Halle-Wittenberg, Wolfgang-Langenbeck-Str. 4, 06120 Halle (Saale), Germany; b Institute of Molecular Biology "Roumen Tsanev", Bulgarian Academy of Sciences, Acad. G. Bonchev Str., Bl. 21, Sofia 1113, Bulgaria; Vienna University of Technology, Austria

**Keywords:** crystal structure, 4-picolyl­amine, perchlorate, conformation, heterocycle, hydrogen bonding

## Abstract

Pyridin-4-ylmethanaminium perchlorate monohydrate (synonym: 4-picolyl­ammonium perchlorate monohydrate), C_6_H_9_N_2_^+^·ClO_4_^−^·H_2_O, crystallizes in the monoclinic system (space group *P*2_1_/*n*) with the asymmetric unit comprising two formula units (*Z*′ = 2). All mol­ecular entities are located on general positions. The two crystallographically distinct 4-picolyl­ammonium cations exhibit different conformations. The two unique perchlorate anions are non-disordered, showing an r.m.s. deviation of 0.011 Å from mol­ecular *T*_d_ symmetry. The supra­molecular structure in the solid state features an intricate tri-periodic network of N—H⋯O, O—H⋯N and O—H⋯O hydrogen bonds.

## Abstract 2

The title compound crystallizes in the monoclinic space group *P*2_1_/*n* with two formula units per asymmetric unit (*Z*′ = 2) and features an intricate tri-periodic hydrogen-bonding network.

## Graphical abstract

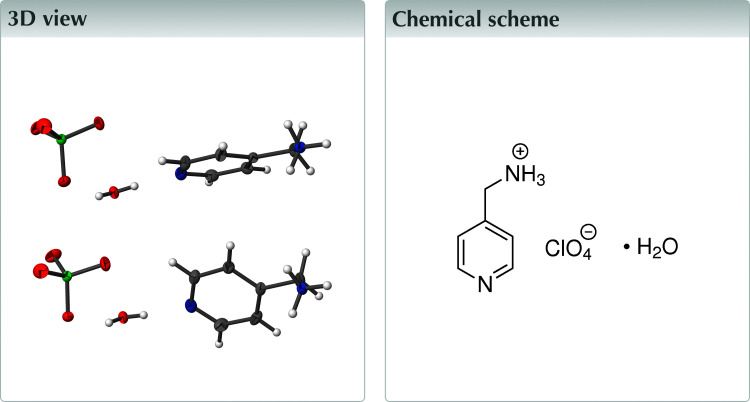

## Structure description

The number of structurally characterized 1:1 salts of the feedstock chemical 4-picolyl­amine is limited. A search of the Cambridge Structural Database (CSD, version 5.43 with November 2022 updates; Groom *et al.*, 2016 ▸) revealed nine crystal structures: the hydrogen chloride (CSD refcode: QANWOS; de Vries *et al.*, 2005 ▸) and hydrogen bromide (TENDUP; Zuffa *et al.*, 2023 ▸), substituted benzoic acid salts (TOHYEV, TOHYIZ; Lemmerer *et al.*, 2008 ▸ and WEBXAE; Ding *et al.*, 2012 ▸), group 10 tetra­cyanidometallates (OFEWUT, OFEXII and OFEXUU; Karaağaç *et al.*, 2013 ▸) and a deca­vanadate (HEBJOR; Msaadi *et al.*, 2022 ▸). We herein report the crystal structure of the monohydrate of the perchlorate salt of 4-picolyl­amine, (**1**).

**Figure 1:**
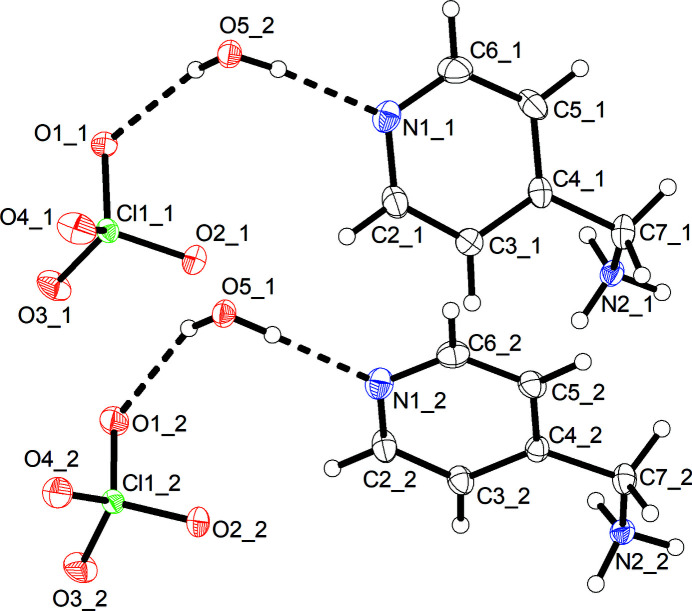
Asymmetric unit of (**1**). Displacement ellipsoids are drawn at the 50% probability level. Hydrogen atoms are represented by small spheres of arbitrary radius. The number after the underscore indicates unique mol­ecules 1 and 2 in each case. Dashed lines represent O—H⋯O and O—H⋯N hydrogen bonds.

As shown in Fig. 1 ▸, the asymmetric unit of (**1**) comprises two formula units C_6_H_9_N_2_^+^ClO_4_^−^·H_2_O (*Z*′ = 2). The amino group of 4-picolyl­amine, which is the more basic site (p*K*_a_ = 8.30; Milletti *et al.*, 2010 ▸) compared to the pyridine nitro­gen atom, is in a protonated state. The two crystallographically distinct 4-picolyl­ammonium cations differ in their conformations. The C3—C4—C7—N2 torsion angle is 67.4 (3)° in mol­ecule 1 and 13.2 (3)° in mol­ecule 2. The difference is ascribable to inter­molecular inter­actions and packing effects in the solid state. In the nine crystal structures containing 4-picolyl­ammonium ions deposited with the CSD, the torsion angles range from 6.4° in HEBJOR to 88.5° in WEBXAE, indicating great conformational flexibility. The mol­ecular structure of cation 2 in (**1**) exhibits an r.m.s. deviation from *C*_S_ point group symmetry of 0.082 Å, as calculated with MOLSYM in *PLATON* (Spek, 2020 ▸). The two crystallographically distinct perchlorate anions are non-disordered, both showing an r.m.s. deviation of 0.011 Å from mol­ecular *T*_d_ point group symmetry.

**Figure 2:**
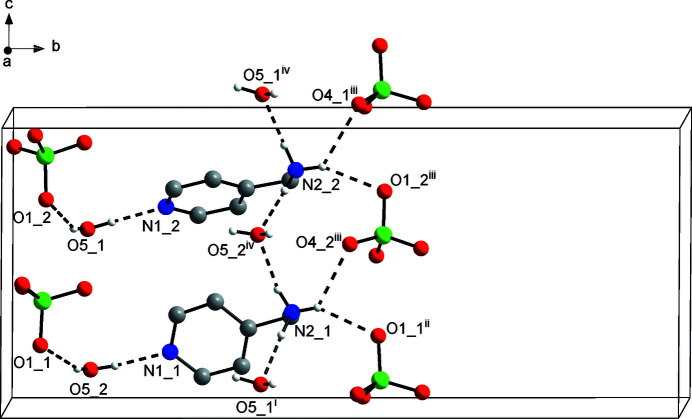
Part of the crystal structure of (**1**) viewed approximately along the *a*-axis direction towards the origin, showing N—H⋯O, O—H⋯N and O—H⋯O hydrogen bonds (dashed lines). The number after the underscore indicates unique mol­ecules 1 and 2 in each case. Carbon-bound hydrogen atoms are omitted for clarity. Symmetry codes refer to Table 1 ▸.

Apart from Coulombic inter­actions, the supra­molecular structure in (**1**) is dominated by classical N—H⋯O, O—H⋯N and O—H⋯O hydrogen bonds. Fig. 2 ▸ depicts a part of the crystal structure, illustrating the crystallographically unique hydrogen bonds. As hydrogen-bond donors, the water mol­ecules join the 4-picolyl­ammonium and perchlorate ions through O—H⋯N_pyridine_ and O—H⋯O hydrogen bonds, respectively. Towards the protonated amino groups, the water mol­ecules act as hydrogen-bond acceptors for N—H⋯O hydrogen bonds, resulting in hydrogen-bonded chains propagating parallel to the *c*-axis direction. The remaining hydrogen-bond donor sites of the 4-picolyl­ammonium ions form donating bifurcated N—H⋯O hydrogen bonds to perchlorate oxygen atoms, resulting in an intricate tri-periodic network. Table 1 ▸ lists numerical details of the relevant hydrogen bonds in (**1**), which are characteristic of strong hydrogen bonds (Thakuria *et al.*, 2017 ▸).

## Synthesis and crystallization

Compound (**1**) was synthesized by adding a solution of 4-picolyl­amine (216 mg, 2 mmol) in 40 ml of ethanol to 40 ml of 0.1 *M* perchloric acid. The reaction mixture was stirred for 4 h at room temperature and then left at ambient conditions. After one week, the precipitate was collected by filtration and air-dried. Colourless crystals of (**1**) suitable for X-ray diffraction were grown from a methanol/water solution at room temperature over a period of three weeks, while the solvents were allowed to evaporate slowly. *Caution:* organic perchlorate salts are potentially explosive and should be handled with care!

## Refinement

Crystal data, data collection and structure refinement details are listed in Table 2 ▸.

## Supplementary Material

Crystal structure: contains datablock(s) I, global. DOI: 10.1107/S2414314623004595/wm4189sup1.cif

Structure factors: contains datablock(s) I. DOI: 10.1107/S2414314623004595/wm4189Isup2.hkl

Supporting information file. DOI: 10.1107/S2414314623004595/wm4189Isup3.cdx

Supporting information file. DOI: 10.1107/S2414314623004595/wm4189Isup4.cml

CCDC reference: 2265167

Additional supporting information:  crystallographic information; 3D view; checkCIF report

## Figures and Tables

**Table 1:** Hydrogen-bond geometry (Å, °)

*D*—H⋯*A*	*D*—H	H⋯*A*	*D*⋯*A*	*D*—H⋯*A*
N2_1—H2*A*_1⋯O5_1^i^	0.90 (2)	1.96 (2)	2.862 (3)	176 (2)
N2_1—H2*B*_1⋯O1_1^ii^	0.90 (2)	2.20 (2)	2.916 (2)	136 (2)
N2_1—H2*B*_1⋯O4_2^iii^	0.90 (2)	2.32 (2)	2.924 (2)	125 (2)
N2_1—H2*C*_1⋯O5_2^iv^	0.90 (2)	1.95 (2)	2.838 (3)	168 (2)
O5_1—H5*A*_1⋯N1_2	0.84 (2)	1.91 (2)	2.751 (2)	176 (3)
O5_1—H5*B*_1⋯O1_2	0.83 (2)	2.21 (2)	2.986 (2)	156 (3)
N2_2—H2*A*_2⋯O5_1^iv^	0.92 (2)	1.92 (2)	2.839 (3)	173 (2)
N2_2—H2*B*_2⋯O4_1^iii^	0.91 (2)	2.42 (2)	3.079 (3)	130 (2)
N2_2—H2*B*_2⋯O1_2^iii^	0.91 (2)	2.19 (2)	2.979 (2)	144 (2)
N2_2—H2*C*_2⋯O5_2^iv^	0.90 (2)	1.98 (2)	2.872 (3)	177 (2)
O5_2—H5*A*_2⋯N1_1	0.83 (2)	1.93 (2)	2.761 (2)	175 (3)
O5_2—H5*B*_2⋯O1_1	0.83 (2)	2.09 (2)	2.874 (2)	160 (3)

Symmetry codes: (i) 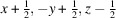 ; (ii) 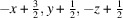 ; (iii) 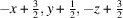 ; (iv) 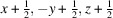 .

**Table 2:** Experimental details

Crystal data	
Chemical formula	C_6_H_9_N_2_^+^·ClO_4_^−^·H_2_O
*M*_r_	226.62
Crystal system, space group	Monoclinic, *P*2_1_/*n*
Temperature (K)	110
*a*, *b*, *c* (Å)	9.1239 (2), 22.1397 (6), 9.5463 (3)
β (°)	101.799 (3)
*V* (Å^3^)	1887.62 (9)
*Z*	8
Radiation type	Mo *K*α
μ (mm^−1^)	0.41
Crystal size (mm)	0.28 × 0.20 × 0.10

Data collection	
Diffractometer	Xcalibur2, Oxford Diffraction
Absorption correction	Multi-scan (*ABSPACK* in *CrysAlis PRO*; Rigaku OD, 2022 ▸)
*T*_min_, *T*_max_	0.894, 1.000
No. of measured, independent and observed [*I* > 2σ(*I*)] reflections	16791, 4422, 3163
*R*_int_	0.041
(sin θ/λ)_max_ (Å^−1^)	0.679

Refinement	
*R*[*F* ^2^ > 2σ(*F* ^2^)], *wR*(*F* ^2^), *S*	0.043, 0.101, 1.04
No. of reflections	4422
No. of parameters	299
No. of restraints	10
H-atom treatment	H atoms treated by a mixture of independent and constrained refinement
Δρ_max_, Δρ_min_ (e Å^−3^)	0.44, −0.42

Computer programs: *CrysAlis PRO* (Rigaku OD, 2022 ▸), *SHELXS* (Sheldrick, 2008 ▸), *SHELXL* (Sheldrick, 2015 ▸), *DIAMOND* (Brandenburg, 2018 ▸) and *publCIF* (Westrip, 2010 ▸).

## full crystallographic data

### Crystal data

**Table:**

C_6_H_9_N_2_^+^·ClO_4_^−^·H_2_O	*F*(000) = 944
*M**_r_* = 226.62	*D*_x_ = 1.595 Mg m^−^^3^
Monoclinic, *P*2_1_/*n*	Mo *K*α radiation, λ = 0.71073 Å
*a* = 9.1239 (2) Å	Cell parameters from 4316 reflections
*b* = 22.1397 (6) Å	θ = 3.6–28.4°
*c* = 9.5463 (3) Å	µ = 0.41 mm^−^^1^
β = 101.799 (3)°	*T* = 110 K
*V* = 1887.62 (9) Å^3^	Prism, colourless
*Z* = 8	0.28 × 0.20 × 0.10 mm

### Data collection

**Table:**

Xcalibur2, Oxford Diffraction diffractometer	4422 independent reflections
Radiation source: fine-focus sealed X-ray tube, Enhance (Mo) X-ray Source	3163 reflections with *I* > 2σ(*I*)
Graphite monochromator	*R*_int_ = 0.041
Detector resolution: 8.4171 pixels mm^-1^	θ_max_ = 28.9°, θ_min_ = 2.8°
ω scans	*h* = −12→11
Absorption correction: multi-scan (*ABSPACK* in *CrysAlisPro*; Rigaku OD, 2022)	*k* = −29→27
*T*_min_ = 0.894, *T*_max_ = 1.000	*l* = −11→12
16791 measured reflections	

### Refinement

**Table:**

Refinement on *F*^2^	Primary atom site location: structure-invariant direct methods
Least-squares matrix: full	Secondary atom site location: difference Fourier map
*R*[*F*^2^ > 2σ(*F*^2^)] = 0.043	Hydrogen site location: mixed
*wR*(*F*^2^) = 0.101	H atoms treated by a mixture of independent and constrained refinement
*S* = 1.04	*w* = 1/[σ^2^(*F*_o_^2^) + (0.0371*P*)^2^ + 1.2652*P*] where *P* = (*F*_o_^2^ + 2*F*_c_^2^)/3
4422 reflections	(Δ/σ)_max_ < 0.001
299 parameters	Δρ_max_ = 0.44 e Å^−^^3^
10 restraints	Δρ_min_ = −0.42 e Å^−^^3^

### Special details

**Table:**

Geometry. All esds (except the esd in the dihedral angle between two l.s. planes) are estimated using the full covariance matrix. The cell esds are taken into account individually in the estimation of esds in distances, angles and torsion angles; correlations between esds in cell parameters are only used when they are defined by crystal symmetry. An approximate (isotropic) treatment of cell esds is used for estimating esds involving l.s. planes.
Refinement. Nitrogen-bound and water hydrogen atoms were located from difference-Fourier maps and were refined with N—H and O—H distances restrained to target values of 0.91 (2) and 0.84 (2) Å, respectively. The respective *U*_iso_(H) values were refined freely.

### Fractional atomic coordinates and isotropic or equivalent isotropic displacement parameters (Å^2^)

**Table:**

	*x*	*y*	*z*	*U*_iso_*/*U*_eq_	
C2_1	0.5915 (3)	0.25285 (10)	0.3275 (3)	0.0236 (5)	
H2_1	0.613952	0.219921	0.392116	0.028*	
C3_1	0.6384 (3)	0.30978 (10)	0.3757 (3)	0.0211 (5)	
H3_1	0.691704	0.315629	0.471201	0.025*	
C4_1	0.6065 (2)	0.35838 (9)	0.2826 (2)	0.0173 (5)	
C5_1	0.5272 (3)	0.34736 (10)	0.1463 (3)	0.0235 (5)	
H5_1	0.501877	0.379550	0.080013	0.028*	
C6_1	0.4846 (3)	0.28865 (11)	0.1070 (3)	0.0260 (6)	
H6_1	0.429816	0.281688	0.012509	0.031*	
C7_1	0.6564 (3)	0.42157 (10)	0.3275 (3)	0.0206 (5)	
H7A_1	0.610755	0.450463	0.251989	0.025*	
H7B_1	0.622219	0.432207	0.416360	0.025*	
N1_1	0.5162 (2)	0.24152 (8)	0.1945 (2)	0.0214 (4)	
N2_1	0.8230 (2)	0.42629 (9)	0.3526 (2)	0.0171 (4)	
H2A_1	0.854 (3)	0.4114 (11)	0.276 (2)	0.027 (7)*	
H2B_1	0.853 (3)	0.4645 (8)	0.371 (3)	0.020 (6)*	
H2C_1	0.861 (3)	0.4058 (11)	0.434 (2)	0.034 (8)*	
Cl1_1	0.71953 (6)	0.05644 (2)	0.38455 (5)	0.01373 (13)	
O1_1	0.70326 (17)	0.05322 (6)	0.23033 (16)	0.0180 (3)	
O2_1	0.7178 (2)	0.11825 (7)	0.42615 (17)	0.0287 (4)	
O3_1	0.85768 (17)	0.02837 (7)	0.45115 (18)	0.0251 (4)	
O4_1	0.59712 (18)	0.02479 (8)	0.42438 (18)	0.0268 (4)	
O5_1	0.43372 (18)	0.11734 (7)	0.61174 (17)	0.0181 (3)	
H5A_1	0.453 (3)	0.1534 (9)	0.638 (3)	0.041 (9)*	
H5B_1	0.513 (2)	0.0983 (12)	0.620 (3)	0.042 (9)*	
C2_2	0.6473 (3)	0.24806 (10)	0.7655 (3)	0.0221 (5)	
H2_2	0.716822	0.216114	0.792595	0.027 (7)*	
C3_2	0.6939 (3)	0.30644 (10)	0.8024 (2)	0.0195 (5)	
H3_2	0.792866	0.313941	0.853765	0.026 (7)*	
C4_2	0.5946 (2)	0.35391 (9)	0.7636 (2)	0.0160 (5)	
C5_2	0.4506 (3)	0.33949 (10)	0.6910 (2)	0.0202 (5)	
H5_2	0.378075	0.370385	0.663996	0.020 (6)*	
C6_2	0.4143 (3)	0.28017 (10)	0.6587 (2)	0.0218 (5)	
H6_2	0.315601	0.271289	0.608434	0.025 (7)*	
C7_2	0.6330 (2)	0.41931 (10)	0.7943 (3)	0.0193 (5)	
H7A_2	0.594521	0.443333	0.707070	0.018 (6)*	
H7B_2	0.581862	0.433608	0.870188	0.022 (6)*	
N1_2	0.5099 (2)	0.23416 (8)	0.6940 (2)	0.0218 (4)	
N2_2	0.7960 (2)	0.43016 (9)	0.8405 (2)	0.0175 (4)	
H2A_2	0.833 (3)	0.4138 (10)	0.9295 (19)	0.022 (6)*	
H2B_2	0.818 (3)	0.4699 (8)	0.854 (3)	0.028 (7)*	
H2C_2	0.841 (3)	0.4149 (11)	0.773 (2)	0.033 (8)*	
Cl1_2	0.72640 (6)	0.05939 (2)	0.88521 (5)	0.01431 (13)	
O1_2	0.72928 (19)	0.06090 (7)	0.73443 (17)	0.0231 (4)	
O2_2	0.67297 (19)	0.11607 (7)	0.92682 (17)	0.0224 (4)	
O3_2	0.87356 (17)	0.04688 (7)	0.96603 (18)	0.0249 (4)	
O4_2	0.62605 (18)	0.01185 (7)	0.90900 (17)	0.0234 (4)	
O5_2	0.43738 (18)	0.12300 (7)	0.12765 (17)	0.0181 (3)	
H5A_2	0.458 (3)	0.1593 (8)	0.142 (3)	0.044 (9)*	
H5B_2	0.518 (2)	0.1049 (12)	0.137 (3)	0.049 (10)*	

### Atomic displacement parameters (Å^2^)

**Table:**

	*U*^11^	*U*^22^	*U*^33^	*U*^12^	*U*^13^	*U*^23^
C2_1	0.0233 (12)	0.0177 (11)	0.0287 (14)	0.0016 (10)	0.0028 (10)	0.0043 (10)
C3_1	0.0231 (13)	0.0192 (11)	0.0199 (12)	0.0000 (10)	0.0016 (10)	0.0005 (9)
C4_1	0.0121 (10)	0.0149 (11)	0.0257 (13)	0.0010 (9)	0.0056 (9)	0.0017 (9)
C5_1	0.0205 (12)	0.0223 (12)	0.0253 (13)	−0.0012 (10)	−0.0009 (10)	0.0089 (10)
C6_1	0.0226 (13)	0.0297 (13)	0.0227 (13)	−0.0063 (11)	−0.0024 (10)	0.0006 (10)
C7_1	0.0164 (11)	0.0143 (11)	0.0321 (14)	0.0005 (9)	0.0070 (10)	−0.0004 (9)
N1_1	0.0172 (10)	0.0190 (10)	0.0279 (12)	−0.0034 (8)	0.0042 (9)	−0.0031 (8)
N2_1	0.0198 (10)	0.0133 (10)	0.0180 (11)	−0.0027 (8)	0.0036 (8)	−0.0018 (8)
Cl1_1	0.0121 (2)	0.0130 (2)	0.0158 (3)	0.0005 (2)	0.00203 (19)	0.0011 (2)
O1_1	0.0223 (8)	0.0173 (8)	0.0146 (8)	0.0006 (7)	0.0041 (7)	0.0003 (6)
O2_1	0.0453 (11)	0.0146 (8)	0.0230 (9)	0.0030 (8)	−0.0003 (8)	−0.0031 (7)
O3_1	0.0149 (8)	0.0331 (10)	0.0259 (9)	0.0092 (7)	0.0004 (7)	0.0041 (7)
O4_1	0.0186 (9)	0.0366 (10)	0.0254 (10)	−0.0081 (8)	0.0048 (7)	0.0079 (8)
O5_1	0.0170 (8)	0.0149 (8)	0.0217 (9)	0.0018 (7)	0.0021 (7)	−0.0022 (7)
C2_2	0.0209 (12)	0.0160 (11)	0.0289 (14)	0.0022 (10)	0.0042 (10)	0.0025 (10)
C3_2	0.0158 (11)	0.0180 (11)	0.0238 (13)	0.0006 (9)	0.0017 (10)	0.0022 (9)
C4_2	0.0185 (11)	0.0142 (10)	0.0163 (11)	0.0000 (9)	0.0058 (9)	0.0001 (8)
C5_2	0.0169 (11)	0.0227 (12)	0.0202 (12)	0.0060 (10)	0.0017 (9)	0.0001 (9)
C6_2	0.0164 (12)	0.0274 (12)	0.0207 (13)	−0.0020 (10)	0.0018 (10)	−0.0019 (10)
C7_2	0.0161 (11)	0.0153 (11)	0.0262 (13)	0.0016 (9)	0.0041 (10)	0.0000 (9)
N1_2	0.0210 (10)	0.0193 (10)	0.0255 (11)	−0.0026 (8)	0.0054 (9)	−0.0006 (8)
N2_2	0.0223 (11)	0.0145 (10)	0.0157 (10)	−0.0026 (8)	0.0038 (8)	0.0000 (8)
Cl1_2	0.0141 (3)	0.0136 (2)	0.0150 (3)	0.0009 (2)	0.00248 (19)	0.0008 (2)
O1_2	0.0318 (10)	0.0214 (8)	0.0173 (9)	0.0018 (7)	0.0078 (7)	0.0028 (7)
O2_2	0.0278 (9)	0.0155 (8)	0.0231 (9)	0.0072 (7)	0.0034 (7)	−0.0020 (7)
O3_2	0.0139 (8)	0.0318 (9)	0.0269 (9)	0.0061 (7)	−0.0010 (7)	0.0015 (7)
O4_2	0.0250 (9)	0.0202 (8)	0.0245 (9)	−0.0084 (7)	0.0040 (7)	0.0029 (7)
O5_2	0.0176 (9)	0.0147 (8)	0.0214 (9)	0.0007 (7)	0.0022 (7)	−0.0013 (7)

### Geometric parameters (Å, º)

**Table:**

C2_1—N1_1	1.338 (3)	C2_2—N1_2	1.335 (3)
C2_1—C3_1	1.380 (3)	C2_2—C3_2	1.384 (3)
C2_1—H2_1	0.9500	C2_2—H2_2	0.9500
C3_1—C4_1	1.388 (3)	C3_2—C4_2	1.387 (3)
C3_1—H3_1	0.9500	C3_2—H3_2	0.9500
C4_1—C5_1	1.375 (3)	C4_2—C5_2	1.391 (3)
C4_1—C7_1	1.506 (3)	C4_2—C7_2	1.504 (3)
C5_1—C6_1	1.386 (3)	C5_2—C6_2	1.374 (3)
C5_1—H5_1	0.9500	C5_2—H5_2	0.9500
C6_1—N1_1	1.331 (3)	C6_2—N1_2	1.339 (3)
C6_1—H6_1	0.9500	C6_2—H6_2	0.9500
C7_1—N2_1	1.493 (3)	C7_2—N2_2	1.482 (3)
C7_1—H7A_1	0.9900	C7_2—H7A_2	0.9900
C7_1—H7B_1	0.9900	C7_2—H7B_2	0.9900
N2_1—H2A_1	0.900 (16)	N2_2—H2A_2	0.921 (16)
N2_1—H2B_1	0.895 (16)	N2_2—H2B_2	0.907 (16)
N2_1—H2C_1	0.903 (17)	N2_2—H2C_2	0.897 (17)
Cl1_1—O2_1	1.4260 (16)	Cl1_2—O2_2	1.4316 (15)
Cl1_1—O3_1	1.4323 (16)	Cl1_2—O3_2	1.4322 (16)
Cl1_1—O4_1	1.4341 (16)	Cl1_2—O4_2	1.4431 (16)
Cl1_1—O1_1	1.4508 (15)	Cl1_2—O1_2	1.4454 (16)
O5_1—H5A_1	0.843 (17)	O5_2—H5A_2	0.831 (17)
O5_1—H5B_1	0.827 (17)	O5_2—H5B_2	0.825 (17)
			
N1_1—C2_1—C3_1	123.5 (2)	N1_2—C2_2—C3_2	123.6 (2)
N1_1—C2_1—H2_1	118.2	N1_2—C2_2—H2_2	118.2
C3_1—C2_1—H2_1	118.2	C3_2—C2_2—H2_2	118.2
C2_1—C3_1—C4_1	119.0 (2)	C2_2—C3_2—C4_2	119.3 (2)
C2_1—C3_1—H3_1	120.5	C2_2—C3_2—H3_2	120.3
C4_1—C3_1—H3_1	120.5	C4_2—C3_2—H3_2	120.3
C5_1—C4_1—C3_1	118.0 (2)	C3_2—C4_2—C5_2	117.2 (2)
C5_1—C4_1—C7_1	120.3 (2)	C3_2—C4_2—C7_2	124.3 (2)
C3_1—C4_1—C7_1	121.7 (2)	C5_2—C4_2—C7_2	118.44 (19)
C4_1—C5_1—C6_1	119.1 (2)	C6_2—C5_2—C4_2	119.4 (2)
C4_1—C5_1—H5_1	120.4	C6_2—C5_2—H5_2	120.3
C6_1—C5_1—H5_1	120.4	C4_2—C5_2—H5_2	120.3
N1_1—C6_1—C5_1	123.5 (2)	N1_2—C6_2—C5_2	123.7 (2)
N1_1—C6_1—H6_1	118.2	N1_2—C6_2—H6_2	118.1
C5_1—C6_1—H6_1	118.2	C5_2—C6_2—H6_2	118.1
N2_1—C7_1—C4_1	110.46 (18)	N2_2—C7_2—C4_2	113.15 (18)
N2_1—C7_1—H7A_1	109.6	N2_2—C7_2—H7A_2	108.9
C4_1—C7_1—H7A_1	109.6	C4_2—C7_2—H7A_2	108.9
N2_1—C7_1—H7B_1	109.6	N2_2—C7_2—H7B_2	108.9
C4_1—C7_1—H7B_1	109.6	C4_2—C7_2—H7B_2	108.9
H7A_1—C7_1—H7B_1	108.1	H7A_2—C7_2—H7B_2	107.8
C6_1—N1_1—C2_1	116.9 (2)	C2_2—N1_2—C6_2	116.7 (2)
C7_1—N2_1—H2A_1	109.1 (17)	C7_2—N2_2—H2A_2	111.7 (16)
C7_1—N2_1—H2B_1	111.0 (16)	C7_2—N2_2—H2B_2	112.4 (17)
H2A_1—N2_1—H2B_1	112 (2)	H2A_2—N2_2—H2B_2	102 (2)
C7_1—N2_1—H2C_1	107.8 (18)	C7_2—N2_2—H2C_2	108.0 (18)
H2A_1—N2_1—H2C_1	112 (2)	H2A_2—N2_2—H2C_2	112 (2)
H2B_1—N2_1—H2C_1	105 (2)	H2B_2—N2_2—H2C_2	110 (2)
O2_1—Cl1_1—O3_1	110.58 (10)	O2_2—Cl1_2—O3_2	110.79 (10)
O2_1—Cl1_1—O4_1	109.98 (11)	O2_2—Cl1_2—O4_2	109.43 (10)
O3_1—Cl1_1—O4_1	109.43 (10)	O3_2—Cl1_2—O4_2	109.22 (10)
O2_1—Cl1_1—O1_1	108.97 (9)	O2_2—Cl1_2—O1_2	109.43 (9)
O3_1—Cl1_1—O1_1	109.10 (9)	O3_2—Cl1_2—O1_2	109.58 (10)
O4_1—Cl1_1—O1_1	108.75 (10)	O4_2—Cl1_2—O1_2	108.36 (10)
H5A_1—O5_1—H5B_1	109 (3)	H5A_2—O5_2—H5B_2	107 (3)
			
C3_1—C4_1—C7_1—N2_1	67.4 (3)	C3_2—C4_2—C7_2—N2_2	13.2 (3)

### Hydrogen-bond geometry (Å, º)

**Table:**

*D*—H···*A*	*D*—H	H···*A*	*D*···*A*	*D*—H···*A*
N2_1—H2*A*_1···O5_1^i^	0.90 (2)	1.96 (2)	2.862 (3)	176 (2)
N2_1—H2*B*_1···O1_1^ii^	0.90 (2)	2.20 (2)	2.916 (2)	136 (2)
N2_1—H2*B*_1···O4_2^iii^	0.90 (2)	2.32 (2)	2.924 (2)	125 (2)
N2_1—H2*B*_1···O4_2^i^	0.90 (2)	2.50 (2)	3.034 (3)	119 (2)
N2_1—H2*C*_1···O5_2^iv^	0.90 (2)	1.95 (2)	2.838 (3)	168 (2)
O5_1—H5*A*_1···N1_2	0.84 (2)	1.91 (2)	2.751 (2)	176 (3)
O5_1—H5*B*_1···O1_2	0.83 (2)	2.21 (2)	2.986 (2)	156 (3)
N2_2—H2*A*_2···O5_1^iv^	0.92 (2)	1.92 (2)	2.839 (3)	173 (2)
N2_2—H2*B*_2···O4_1^iii^	0.91 (2)	2.42 (2)	3.079 (3)	130 (2)
N2_2—H2*B*_2···O1_2^iii^	0.91 (2)	2.19 (2)	2.979 (2)	144 (2)
N2_2—H2*C*_2···O5_2^iv^	0.90 (2)	1.98 (2)	2.872 (3)	177 (2)
O5_2—H5*A*_2···N1_1	0.83 (2)	1.93 (2)	2.761 (2)	175 (3)
O5_2—H5*B*_2···O1_1	0.83 (2)	2.09 (2)	2.874 (2)	160 (3)

Symmetry codes: (i) *x*+1/2, −*y*+1/2, *z*−1/2; (ii) −*x*+3/2, *y*+1/2, −*z*+1/2; (iii) −*x*+3/2, *y*+1/2, −*z*+3/2; (iv) *x*+1/2, −*y*+1/2, *z*+1/2.
